# Axonal protection by brimonidine with modulation of p62 expression in TNF-induced optic nerve degeneration

**DOI:** 10.1007/s00417-015-3005-3

**Published:** 2015-04-12

**Authors:** Yasushi Kitaoka, Kaori Kojima, Yasunari Munemasa, Kana Sase, Hitoshi Takagi

**Affiliations:** Department of Ophthalmology, St. Marianna University School of Medicine, 2-16-1 Sugao, Miyamae-ku, Kawasaki, Kanagawa 216-8511 Japan; Department of Molecular Neuroscience, St. Marianna University Graduate School of Medicine, 2-16-1 Sugao, Miyamae-ku, Kawasaki, Kanagawa 216-8511 Japan

**Keywords:** p62, Brimonidine, Autophagy, Tumor necrosis factor, Optic nerve

## Abstract

**Purpose:**

The p62, also called sequestosome 1 (SQSTM1), plays a crucial role in tumor necrosis factor (TNF)-induced optic nerve degeneration. Brimonidine has been shown to have protective effects on retinal ganglion cell bodies, although its role in their axons remains to be examined. We determined whether brimonidine modulates axonal loss induced by TNF and affects the expression of p62 in the optic nerve.

**Methods:**

Experiments were performed on adult male Wistar rats that received an intravitreal injection of 10 ng TNF alone or simultaneous injection of TNF and 2, 20, or 200 pmol of brimonidine tartrate. The expression of p62 in the optic nerve was examined by immunoblot analysis. The effects of brimonidine on axons were evaluated by counting axon numbers 2 weeks after intravitreal injection.

**Results:**

Intravitreal injection of brimonidine exerted substantial axonal protection against TNF-induced optic nerve degeneration. Immunoblot analysis showed that p62 was upregulated in the optic nerve after intravitreal injection of TNF, and that this increase was completely inhibited by brimonidine. Treatment with brimonidine alone also significantly decreased p62 protein levels in the optic nerve compared with the basal level.

**Conclusions:**

These results suggest that the modulation of p62 levels in the optic nerve by brimonidine may be involved partly in its axonal protection.

## Introduction

Brimonidine is a α_2_-adrenoreceptor agonist that lowers the intraocular pressure (IOP) and is widely used for glaucoma treatment. Several studies demonstrated that brimonidine has a neuroprotective effect on retinal ganglion cells (RGCs). For example, brimonidine protected RGCs in an in vivo transgenic model of excessive oxidative stress [1] and protected RGCs against ischemia [2, 3]. It was shown that intravitreal injection of brimonidine upregulated brain-derived neurotrophic factor (BDNF) expression in the rat retina [4]. Other reports also demonstrated that brimonidine increased BDNF and p-AKT expression and protected RGCs in the ocular hypertensive rat retina [5]. Moreover, brimonidine inhibited the increases in the expression of mitochondrial transcription factor A and oxidative phosphorylation complex in the ischemic retina [6]. Those studies focused on RGC body protection, and the role brimonidine plays in their axons remains to be examined.

The p62, also called sequestosome 1 (SQSTM1), plays crucial roles in the autophagy machinery, and its accumulation has been linked to neurodegenerative disease [7–9]. Upregulated p62 was found in the compressed spinal cord, and the forced expression of p62 decreased the number of neuronal cells under hypoxic stress [10]. It was demonstrated that the overexpression of p62 promotes apoptosis with the activation of caspase-8, while knockdown of p62 reduces human glioma cell death [11]. We previously found that there was a substantial increase in p62 protein levels in optic nerve samples 1 week after IOP elevation in a rat hypertensive glaucoma model [12]. More recently, we have demonstrated that there was also an increase in p62 protein levels in the optic nerve after intravitreal injection of tumor necrosis factor (TNF) and that inhibition of p62 resulted in axonal protection in the optic nerve [13]. The TNF is involved in certain types of glaucoma [14–19], and the TNF injection model may be useful in understanding the mechanism of axonal degeneration of RGCs [20]. In the present study, we attempted to determine whether brimonidine modulates axonal loss in TNF-induced optic nerve degeneration and affects the expression of p62 in the optic nerve.

## Materials and methods

### Animals

Experiments were performed on 50- to 55-day-old male Wistar rats; 44 rats and 22 rats were used for the immunoblot analysis and axon counting studies, respectively. All studies were conducted according to the Association for Research in Vision and Ophthalmology (ARVO) statement for the Use of Animals in Ophthalmic and Vision Research and were approved by the Ethics Committee of the Institute of Experimental Animals of St. Marianna University Graduate School of Medicine. The animals were housed in controlled conditions, with temperature at 23 ± 1 °C, humidity at 55 ± 5 %, and light from 06:00 to 18:00.

### Administration of TNF

Intravitreal injection of TNF (Sigma-Aldrich, St. Louis, MO, USA) was performed as described previously [20, 21]. Briefly, rats were anesthetized with an intramuscular injection of a mixture of ketamine-xylazine (10 and 4 mg/kg, respectively). A single 2-μl injection of 10 ng of TNF in 0.01 M PBS, pH 7.40, was administered intravitreally into the right eye of an animal under a microscope to avoid lens injury. The PBS alone was administered into the contralateral left eye as a control. In the brimonidine treatment groups, 2, 20, or 200 pmol of brimonidine tartrate (Senju Pharmaceutical Co., Ltd., Osaka, JAPAN) in 0.01 M PBS was mixed with 10 ng of TNF and administered intravitreally in a simultaneous injection. A previous study used a single 5-μl intravitreal injection of brimonidine (0.85 μM to 34 μM, i.e., 4.25 pmol to 170 pmol) in rats and showed the upregulation of BDNF in RGCs [4]. Therefore, our current brimonidine dosage (2-μl intravitreal injection of 1 μM to 100 μM) is likely to be a similar concentration. The rats were euthanized 1 or 2 weeks after the intravitreal injections with an intraperitoneal overdose of sodium pentobarbital, followed by enucleation of the eyes.

### Immunoblot analysis

Forty-four rats were used for immunoblot analysis as described previously [22]. Briefly, 1 or 2 weeks after intravitreal injection, optic nerves (4 mm in length starting immediately behind the globe) were collected, homogenized, and then centrifuged at 15,000 × g for 15 min at 4 °C. Two optic nerve specimens were pooled into one sample, e.g., *n* = 5 included ten independent optic nerve samples per group. Protein concentrations were determined using the Bio-Rad Protein Assay kit (Bio-Rad, Hercules, CA, USA). Protein samples (5 μg per lane) were subjected to SDS-PAGE on gels (Bio-Rad) and transferred to PVDF membranes (Immobilon-P, Millipore, Billerica, MA, USA). Membranes were blocked with Tris-buffered saline (TBS)-0.1 % Tween-20 containing 5 % skim milk. Membranes were first reacted with anti-p62 antibody (1:200; Medical & Biological Laboratories Co., Nagoya, Japan) or anti-β-actin antibody (1:500; Sigma-Aldrich) in TBS containing 5 % skim milk. Membranes were then sequentially exposed to peroxidase-labeled anti-rabbit IgG antibody (Cappel, Solon, OH, USA) or peroxidase-labeled anti-mouse IgG antibody (Cappel) diluted 1:5000 in Tween-20 in TBS. Western blots were visualized with an ECL detection system (Amersham ECL Prime Western Blotting Detection Reagents, GE Healthcare, Buckinghamshire, UK).

### Axon counting in optic nerves

Morphometric analysis of each optic nerve was performed as described previously with samples from 22 rats [20–22]. Eyes were obtained from the animals 2 weeks after intravitreal injection. Four-millimeter segments of the optic nerves were obtained starting 1 mm behind the globe. These segments of optic nerve were fixed by immersion in Karnovsky’s solution for 24 h at 4 °C, processed, and embedded in acrylic resin. Cross sections (1 μm thick) were cut beginning 1 mm from the globe and stained with a solution of 1 % paraphenylene-diamine (Sigma-Aldrich) in absolute methanol. For each section, images at the center and at each quadrant of the periphery (approximately 141.4 μm from the center) were acquired with a light microscope (BX51; Olympus, Tokyo, JAPAN) with a 100× coupled digital camera (MP5Mc/OL; Olympus) and associated QCapture Pro software (version 5.1, QImaging, Surrey, Canada). The acquired images were quantified using Aphelion image-processing software (version 3.2, ADCIS SA and AAI, Inc., Hérouville Saint Clair, France). The number of axons was determined in five distinct areas of 1446.5 μm^2^ each (each quadrant of the periphery in addition to the center; total area of 7232.3 μm^2^ per eye) from each eye. The number of axons per eye was averaged and expressed as the number per square millimeter. A minimum of five eyes per experimental condition was used for analysis.

### Statistical analysis

Data are presented as mean ± SEM. Differences among groups were analyzed using one-way ANOVA, followed by the Mann–Whitney test. A probability value of less than 0.05 was considered to represent a statistically significant difference.

## Results

### Effects of brimonidine on TNF-induced axonal degeneration

**Fig. 1:**
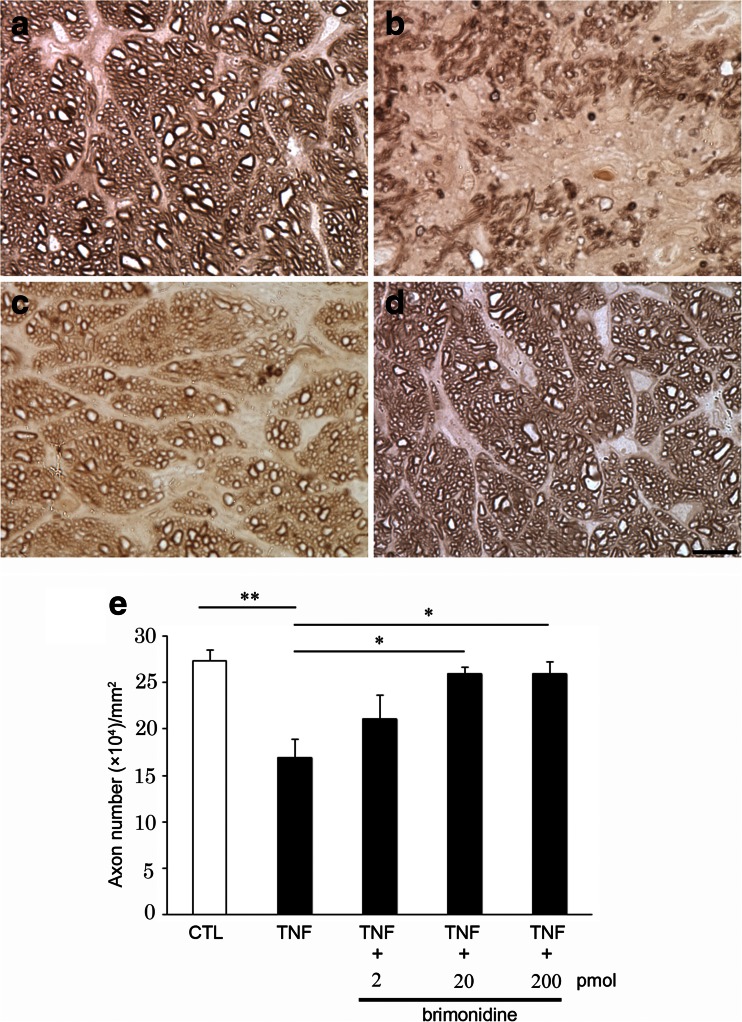
Brimonidine prevented TNF-induced axon loss. Light microscopic findings 2 weeks after (**a**) PBS injection, (**b**) 10-ng TNF injection, (**c**) 10-ng TNF + 20-nmol brimonidine injection, or (**d**) 10-ng TNF +200-nmol brimonidine injection. Scale bar = 10 μm (a–d). (**e**) Effect of brimonidine (2–200 nmol) on axon numbers in the optic nerve. Each column represents mean ± SEM; *n* = 4–7 per group. *p < 0.05; **p < 0.005

We previously demonstrated that after an approximately 30 % loss of axons 2 weeks after TNF injection, there was no further loss of axons at 1 or 2 months [20]. In the present study, compared with PBS-treated eyes (Fig. 1a), we confirmed substantial degenerative changes in the axons 2 weeks after TNF injection (Fig. 1b), which were consistent with the findings of our previous studies [20–22]. Brimonidine 20 pmol-treated eyes showed apparent attenuated effects with better-preserved nerve fibers (Fig. 1c). Substantial protective effects on axons against TNF-induced optic nerve degeneration were also seen in brimonidine 200 pmol-treated eyes (Fig. 1d). Quantitative analysis showed that treatment with brimonidine 2 pmol tended to be modestly protective, but this was not statistically significant (*n* = 4; *p* = 0.1859 versus TNF injection; Fig. 1e). Treatment with brimonidine 20 pmol exerted a significant protective effect against TNF-induced axonal loss (*n* = 4; *p* = 0.00815 versus TNF injection; Fig. 1e). Brimonidine 200 pmol-treated eyes showed 86.4 % axonal protection compared with eyes after TNF injection alone (*n* = 7; *p* = 0.00601 versus TNF injection; Fig. 1e).

### Effects of brimonidine on p62 protein levels in optic nerves

**Fig. 2:**
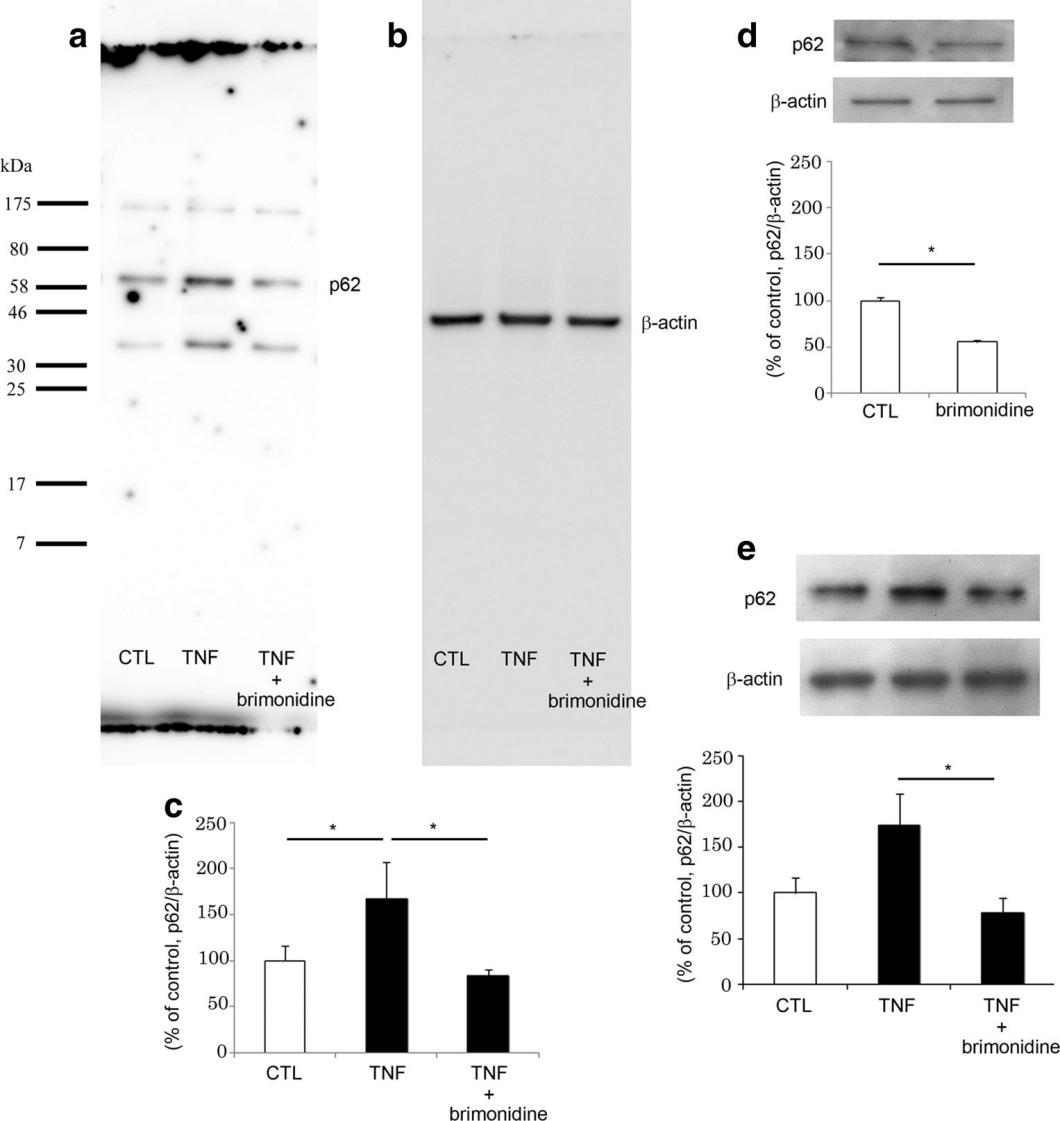
p62 protein levels in optic nerves. Immunoblot data are normalized to β-actin levels in the same sample. **a** Immunoblotting for p62 1 week after PBS injection, 10-ng TNF injection, or 10-ng TNF + 200-nmol brimonidine injection. **b** Immunoblotting for β-actin in the same sample. **c** Data are expressed as percentage of control. Each column represents mean ± SEM. *n* = 5 (10 optic nerves) per group. *p < 0.05. **d** Immunoblotting for p62 1 week after PBS injection or 200-nmol brimonidine injection. Data are expressed as percentage of control. Each column represents mean ± SEM. *n* = eight (8 optic nerves) per group. *p < 0.05. **e** Immunoblotting for p62 2 weeks after PBS injection, 10-ng TNF injection, or 10-ng TNF + 200-nmol brimonidine injection. Data are expressed as percentage of control. Each column represents mean ± SEM. *n* = eight (8 optic nerves) per group. *p < 0.05

We previously found that p62 was increased in optic nerve samples 1 week after TNF injection [13] at the time before axon loss became obvious. In the current study, we examined the effect of brimonidine on p62 protein levels 1 and 2 weeks after intravitreal injection, and the latter time point is when axon loss becomes obvious [20]. Consistent with our previous results [13], there was a significant increase in p62 protein levels in the optic nerve samples 1 week after TNF injection (Fig. 2a–c). This increase was completely abolished by brimonidine (Fig. 2a–c). Moreover, treatment with brimonidine alone significantly decreased p62 protein levels in the optic nerve compared with the basal level (Fig. 2d). Furthermore, there was a tendency for p62 protein levels to increase in the optic nerve samples 2 weeks after TNF injection, but this was not statistically significant (*n* = 4; *p* = 0.083265 versus PBS injection; Fig. 2e). However, this increase was completely abolished by brimonidine (Fig. 2e).

## Discussion

In the present study, brimonidine exerted a substantial protective effect against axonal loss after TNF injection. Axonal protection by brimonidine was also seen in different optic nerve injury models. For example, pretreatment with brimonidine significantly reduced axonal loss in an ischemic optic neuropathy model [23]. This is consistent with the results of a previous study showing that systemic brimonidine, which did not affect IOP, exerted substantial axonal protection following ocular hypertension [24]. That study found that systemic brimonidine ameliorated anterograde transport deficits due to ocular hypertension [24]. Thus, it is possible that the protective effects of brimonidine are associated with its improvement of anterograde and retrograde transport [25, 26]. In addition to axonal protection, axonal regeneration by brimonidine was reported. It was shown that treatment with brimonidine resulted in enhanced axonal growth in juvenile, glaucomatous, and optic nerve crush retinas in vitro [27]. A recent in vivo study has determined that Erk1/2 activity is required for brimonidine-mediated axonal regeneration after optic nerve injury [28]. Thus, it is likely that brimonidine has beneficial effects on axonal protection as well as axonal regeneration in several distinct types of optic nerve damage.

A constitutively high level of p62 under pathological conditions leads to the accumulation of damaged mitochondria and subsequent reactive oxygen species production [29], and the accumulation of p62 after autophagy inhibition causes a delay in the clearance of short-lived ubiquitin-proteasome system-specific substrates, like p53, which may mediate toxicity [30]. In the present study, we observed an increase in the p62 protein level in the optic nerve after TNF injection, consistent with the results of our previous study [13], and this increase was completely inhibited by the simultaneous injection of brimonidine. It is interesting to note that autophagosome formation was increased in response to β_2_-agonist administration in rat skeletal muscle [31]. Augmented formation of autophagosomes was also observed in the K562 cell line following α_1_-agonist treatment [32]. Therefore, it is reasonable to speculate that adrenoceptor stimulation may affect autophagy machinery, although further studies will be needed to clarify the involvement of α-adrenergic receptor regulation within the optic nerve. Because p62 accumulates when autophagy is inhibited, and decreased levels can be observed when autophagy is induced, p62 may be used as a marker of autophagy flux [33]. It was shown that β-adrenoceptor stimulation enhanced autophagic flux by promoting lysosomal degradation in fat cells [34]. It was also reported that norepinephrine strongly enhances autophagic flux in cultured cardiac fibroblasts [35]. The level of p62 may be dependent on the balance between the incoming flux, which is the transcriptional regulation in response to various stimuli, and outgoing flux, which is influenced by autophagic activity [36]. Since we found that brimonidine decreased p62 levels in the optic nerve compared with the basal level, one hypothesis posits that this may be because brimonidine directly affects p62 expression, such as by increasing autophagic flux, rather than indirectly exerting effects resulting from axonal protection. Taking the various results together, it is possible that brimonidine exerts axonal protection with the involvement of autophagy machinery.

In conclusion, the present study showed that the modulation of p62 levels by brimonidine in the optic nerve might be involved in part in its axonal protective effects. Further studies are needed to clarify the mechanism by which brimonidine alters p62 expression.
